# Recurrent respiratory papillomatosis: is patient well-being related to anatomical manifestation?

**DOI:** 10.1007/s00405-026-10016-2

**Published:** 2026-02-06

**Authors:** Constantia J. M. A. Trimbos, Carlijn E. L. Hoekstra, Rico N. P. M. Rinkel, Christine D. L. van Gogh, Birgit I. Lissenberg, Michelle J. Mallinger, Frederik G. Dikkers

**Affiliations:** 1 Department of Otorhinolaryngology, Head and Neck Surgery, Amsterdam UMC location VU University Amsterdam, De Boelelaan 1117, Amsterdam, The Netherlands; 2 Department of Otorhinolaryngology, Head and Neck Surgery, Amsterdam UMC location University of Amsterdam, Meibergdreef 9, Amsterdam, The Netherlands; 3 Department of Epidemiology and Data Science, Amsterdam UMC location VU University Amsterdam, De Boelelaan 1117, Amsterdam, The Netherlands; 4 Department of Otorhinolaryngology, OLVG, Jan Tooropstraat 164, Amsterdam, 1061 AE The Netherlands; 5 Department of Otorhinolaryngology, Head and Neck Surgery, University Medical Center Groningen, Hanzeplein 1, Groningen, 9713 GZ The Netherlands

**Keywords:** RRP, Recurrent respiratory papillomatosis, PROM, Derkay score

## Abstract

**Purpose:**

To date, there is no uniform staging system to quantify the extent of recurrent respiratory papillomatosis (RRP). In scientific research on RRP, patient factor has not been leading in developing its potential staging system. Therefore, this report investigated the association between objective anatomical measurement and Patient Reported Outcome Measures (PROMs).

**Methods:**

The extent of RRP was determined according to the Derkay score. Images from RRP patients were retrieved from laryngoscopic examinations (during phonation and respiration). Four laryngologists assigned Derkay scores independently. The RRP-specific Distress Thermometer & Problem List (DT&PL) was used as PROM. Derkay scores were compared to DT&PL scores using a linear mixed model.

**Results:**

Eighty-eight pairs of images were studied. The linear mixed model analysis resulted in a regression coefficient of 0.54 (*P* < 0.001, 95% CI 0.28–0.80). The average inter-observer class correlation coefficient was 0.95 (95% CI 0.93–0.96).

**Conclusion:**

A moderate association was found between Derkay and DT&PL scores. Considering that RRP is a benign lesion, proper reflection of patient complaints is essential when staging the condition. Future research should consider the significance of the patient’s quality of life to develop a definitive staging method for clinical practice.

## Introduction

Recurrent respiratory papillomatosis (RRP) is a benign lesion of viral origin. However, it is potentially associated with severe morbidity. The condition causes symptoms such as dysphonia as well as dyspnea, as a result of luminal airway obstruction [1]. Due to the heterogeneous clinical presentation of RRP in terms of location and extent, the severity of complaints varies among patients. Over the last few decades, surgical debulking has been the cornerstone of RRP treatment [2]. In recent years, however, several medical modalities have been recognized as possible adjuvant therapy, e.g., cidofovir, bevacizumab, Gardasil^®^ or immunotherapeutics like INO-3107 or avelumab [3–8].

The severity of RRP can be evaluated in various ways. First, disease severity can be assessed by observing the anatomical extent of RRP by laryngoscopic examination. Several methods have been described to score the quantity of RRP [3, 9]. The most frequently used method is the Derkay score, which was developed to objectively and subjectively evaluate a patient´s clinical course and treatment response [9].

Secondly, severity can be described through a perceptual analysis of the voice. To analyze voice function, methods such as the Consensus Auditory-Perceptual Evaluation of Voice (CAPE-V) and the Auditory Perceptual Voice Evaluation by GRBAS (grade, roughness, breathiness, asthenia, strain) are known [10, 11]. Additionally, a more detailed acoustic voice analysis could be performed using a voice range profile.

Lastly, patient-derived information through questionnaires on voice handicap and quality of life can be used to evaluate the impact of RRP. Examples of these are visual analogue scales or Likert scales like the Voice Handicap Inventory (VHI) or the Voice Related Quality of Life (V-RQOL) [12–14].

**Fig. 1:**
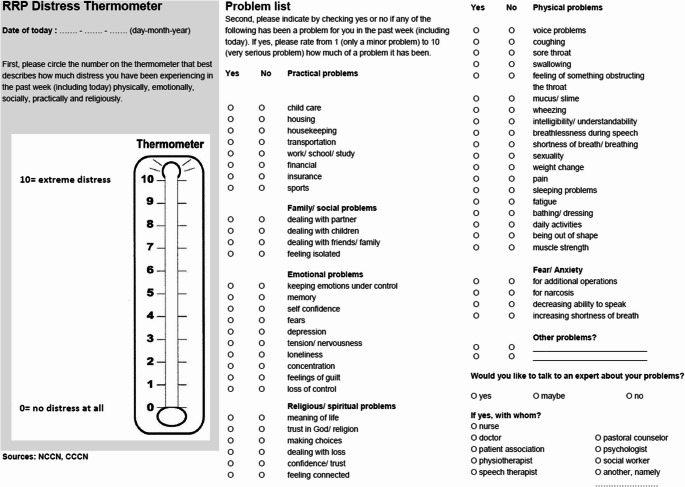
Distress thermometer & problem list

General Quality of life (QoL) can be analyzed using the Distress Thermometer and Problem List (DT&PL) [15]. The RRP-adjusted DT&PL questionnaire is a validated questionnaire for RRP patients. The DT is displayed like a thermometer (Fig. 1). It assesses the patients’ distress on a score ranging from 0 to 10, increasing according to the level of distress. The DT is accompanied by a problem list (PL) with questions to identify the potential origin of distress.

The few studies that have been done on the general QoL of RRP patients show a significantly lower QoL among RRP patients when compared to control groups [16–19]. Likewise, QoL within RRP populations has been described [20]. Besides the clinical complaints due to RRP, like hoarseness, stridor and potential airway compromise, RRP is known for its unpredictable and high recurrence rate and demand for repetitive surgical interventions.

The aim of this study is to evaluate whether the anatomical degree of RRP measured with the Derkay score can predict the patient’s quality of life. This could help determine whether the Derkay score can also be used to support decision-making in clinical practice.

## Materials and methods

### Patient inclusion

Patient data were retrieved from the database of the voice clinic of the Otorhinolaryngology Department of the Amsterdam University Medical Center (a tertiary referral center). This database contains all patients diagnosed with RRP after 2010. Patients were retrospectively included from December 2018 to October 2022. Patients were eligible for inclusion if they had a histologically proven RRP. Data from these patients were included for all their visits within this period if a representative clinical examination video of the entire larynx was available for that visit and if they had a completed distress thermometer and problem list (DT&PL) on the same day. The first author selected the videos. Insufficient video quality led to exclusion. There were no further exclusion criteria.

According to Dutch law patient consent was not required for this study, as anonymous, retrospective data were used in the analysis.

### Subjective assessment by the patient

In this study the DT component of the RRP-adjusted DT&PL questionnaire was utilized as Patient Reported Outcome Measure (PROM) [15]. Patients completed the DT&PL questionnaire prior to consulting the laryngologist.

### Objective assessment

**Fig. 2:**
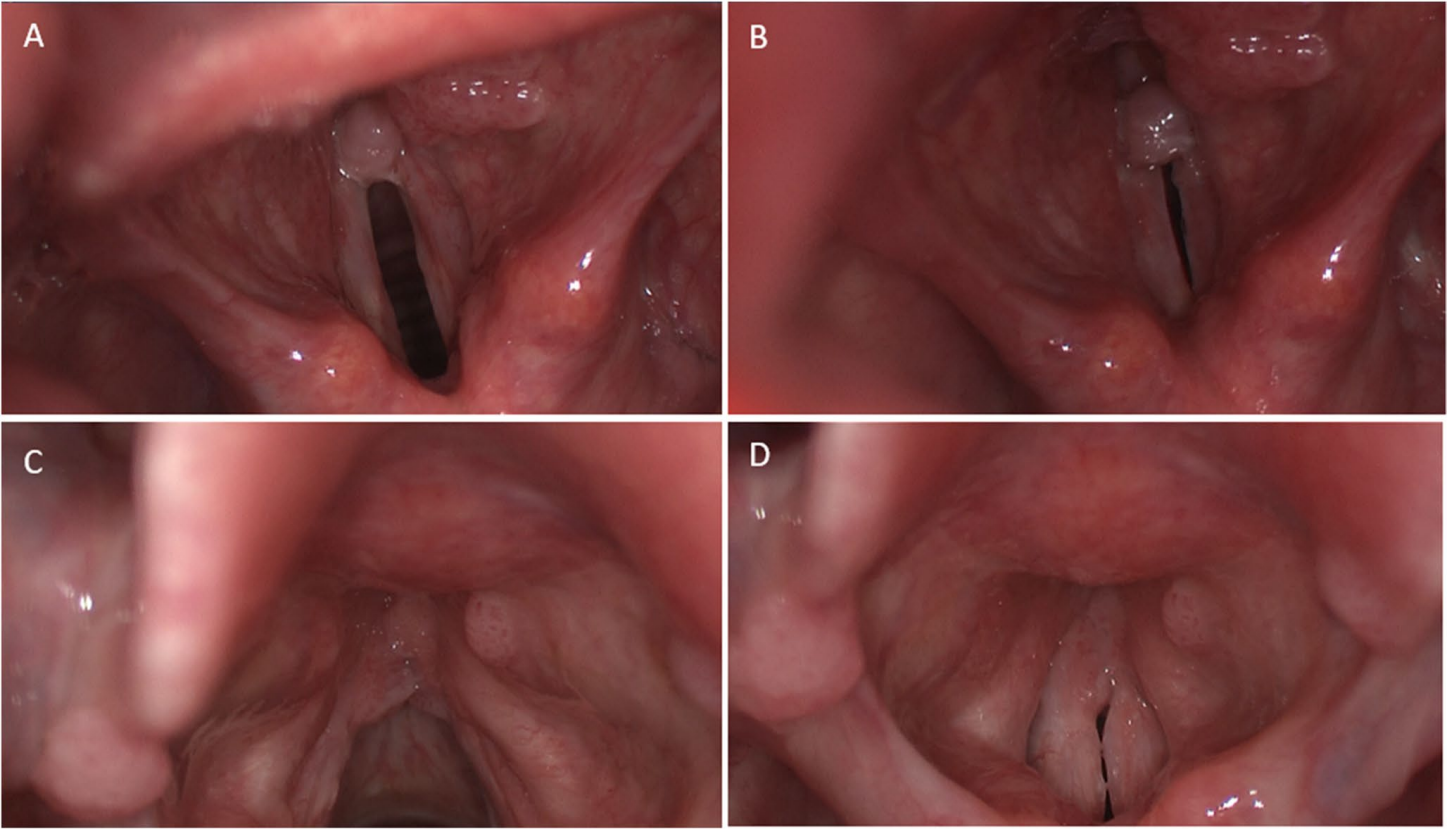
Two pairs of video stills of larynges belonging to patients suffering from recurrent respiratory papillomatosis. **A** case X during respiration; **B** case X during phonation; **C** case Y during respiration; **D** case Y during phonation

Anatomical assessment was performed by scoring laryngeal images using the Derkay score [9]. In the Derkay score, a number (0: none, 1: surface lesion, 2: raised lesion, 3: bulky lesion) is assigned to different epiglottic, laryngeal, and tracheal sites according to the amount of disease. The scores of all sites are summed to a final score. For this study, only the numerical part of the Derkay score was used. Derkay scores were retrospectively assigned to every subject by four independent laryngologists. Their experience as academic laryngologists ranged from 10 to 34 years. Two images from every subject were scored blindly with respect to the patient´s clinical condition. Video examination of the larynx was performed using a 90˚ rigid telescope (Olympus Medical Systems, model WA95105A, Center Valley, PA, United States) on a videolaryngostroboscope (Pentax Medical, Laryngeal Strobe, model 9400, Montvale, NJ, United States). Images were saved on a computer (Pentax Medical, model 9310HD, Montvale, NJ, United States). To stratify the findings of physical examination, two pictures were retrieved from every videolaryngoscopy: one during respiration and the other during phonation. High resolution images were chosen by the main author to clearly depict the extent of the papilloma. Representative images of two subjects are demonstrated in Fig. 2. The order of study subjects was randomized before scoring. Scoring was performed on the same Kay Pentax monitor by all four laryngologists to ensure uniform image quality. One hour was assigned to score all images to make scoring as equal as possible between observers. The calculated mean of the four scores by different laryngologists was then determined to two decimal places for data analysis.

### Statistical analysis

To compare the mean Derkay score with its concurrent DT value, a linear mixed model was used, taking into account the possibility that one patient might be included multiple times with different data. The DT score was set as the outcome. The mean Derkay score was included as a fixed effect and a random intercept was included for the patient. We ensured the reliability of our study by assessing variations in scoring with use of the Derkay score. The inter-observer variability was determined using an inter-class correlation coefficient to objectify the consistency of scoring by different laryngologists. Absolute agreement was estimated with a two-way mixed model. The intra-observer variability was calculated for each observer using an intra-class correlation coefficient. This was executed by duplicating eight inclusions. These duplicates were not included in the overall analysis. All analyses were performed in SPSS version 28 (IBM Corp., Armonk, NY, United States).

## Results

Eighty-eight consultations of 38 different patients were included in the study. Among these patients were 11 females and 27 males, with a mean age of 46.1 years (SD 13.8). The mean age of onset of RRP was 38.6 years (SD 11.9). There were no patients included with Juvenile Onset RRP. 90% of the patients underwent previous surgery for their papillomatosis (median 3.5 surgeries). Eight patients had comorbidities, including cardiovascular disorders, pulmonary disease, kidney insufficiency, diabetes, ulcerative colitis, obstructive sleep apnea syndrome and obesity. All inclusions were typed as HPV-6 or HPV-11 (Table 1).

**Table 1:** Baseline characteristics

Baseline characteristics	*N*	Average	Range
Inclusions, n	88		
Patients	38		
Number of included studies per patient		3	1–6
Sex			
Male	27		
Female	11		
Age			
Age		46.1 years	20–78 years
Age of onset of RRP		38.6 years	11–76 years
Medical history			
HPV 6 or 11	38		
Previous RRP surgery	34		
Number of surgeries		7.3	1–36
Comorbidities			
Cardiovascular disorders	5		
Pulmonary disease	2		
Kidney insufficiency	1		
Diabetes	1		
Ulcerative colitis	1		
Obstructive sleep apnea syndrome	1		
Obesity	1		

DT scores ranged from 0 to 10 (mean 3.5, SD 2.5). Mean Derkay scores ranged from 0 to 12 (mean 3.0, SD 1.8). The mixed model analysis resulted in a regression coefficient of 0.54 (*P* < 0.001, 95% Confidence Interval (CI) 0.28–0.80), showing a significant association between the DT and mean Derkay score.

**Fig. 3:**
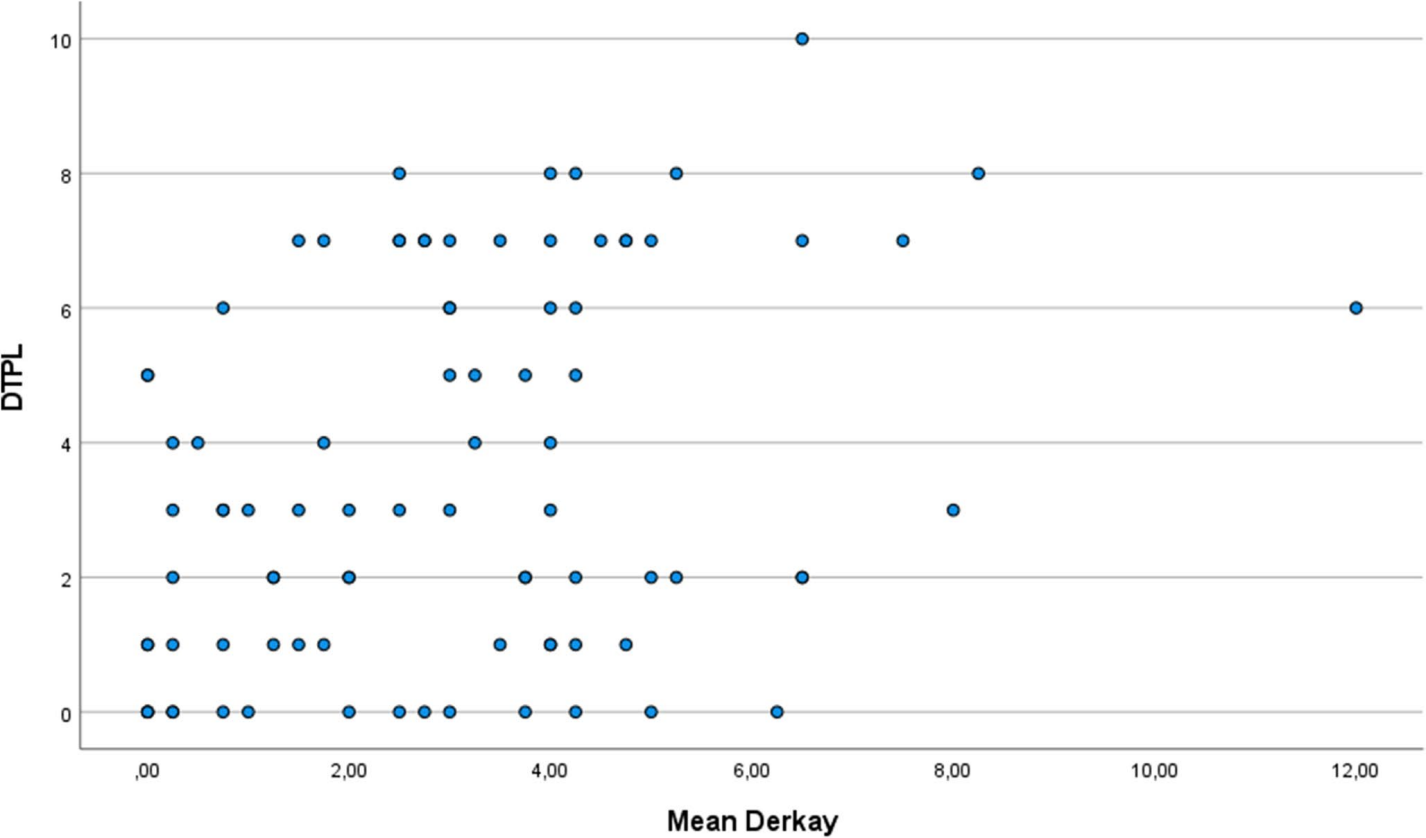
A scatter plot illustrating the correlation between the patient reported DT score from the DT&PL questionnaire and the corresponding Derkay score, which is the average anatomical score provided by four different laryngologists

Fifteen times the Derkay score was ≤ 0.25, with a concurrent DT of 1.6 (range 0–5). Seventeen times the DT score was 0, with a concurrent Derkay score of 2.0 (range 0-6.25). Corresponding Derkay scores to the reported DT are shown in Fig. 3.

In 14 cases (14%) all laryngologists scored identically. In 40 cases (41%) ≥ 3 laryngologists scored identically. The inter-class correlation coefficient was high (ICC = 0.95; 95% CI 0.93–0.96), showing adequate agreement between the reviewers. Eight pairs of images were duplicated and scored a second time. The intra-class correlation coefficient for each observer was 0.84 (95% CI 0.20–0.97), 0.97 (95% CI 0.87–0.96), 0.97 (95% CI 0.85–0.95) and 0.83 (95% CI 0.14–0.97) respectively.

## Discussion

This study analyzed the relationship between RRP expansion using the Derkay score and the patients’ subjective scores on the DT. To the best of our knowledge, this is the first study to evaluate this relationship. A certain conformity was expected between the different domains. It seemed probable that an increase in the Derkay score correlates with greater disease burden.

A statistically significant association was found between Derkay- and concurrent DT scores, but there is only a modest relationship. However, affecting factors must be considered. Potential discrepancy between the two measurements could be explained by the localization of papillomatosis and the origin of complaints. This will be clarified in the following paragraphs.

The quantity of the Derkay score might be related to the extent of papillomatous lesions, but does not necessarily differentiate in the localization of the papilloma. It is important to reconsider this when analyzing patients’ well-being. The score counts all sites of the aerodigestive tract equally, whereas different sites can cause complaints with a contrasting intensity. For example, a bulky papilloma on the laryngeal side of the epiglottis (scored Derkay 3) might cause fewer complaints than a small lesion on the free edge of the true vocal fold (scored Derkay 1). Thus, a higher Derkay score does not automatically imply more complaints. The site of the lesion may represent a potential confounding factor in this study. However, distinguishing between specific subsites could limit the clinical applicability of the findings. Additionally it would result in smaller group sizes that may be insufficient for meaningful statistical analysis.

Furthermore, as part of the DT&PL, the DT has very comprehensive implications. It is a tool to holistically assess the severity and origin of distress in RRP patients [21]. It summarizes various complaints in only one score. The benefit of such a score is that it constitutes a broad clinical applicability. However, its absolute value does not demonstrate its particular origin. Therefore it is not possible to distinguish between complaints caused by RRP manifestation or other underlying issues. The well-known clinical sequelae of surgical interventions in RRP patients are scar formation and laryngeal web. Thus, the number of surgeries in the medical history could also be a potential confounder. These sequelae might negatively influence the DT, while the condition may be in regression at the same time. We considered the DT to be the best-validated instrument for measuring QoL in RRP patients, given its clarity and completeness. Despite the fact that previous questionnaires on voice handicap showed a significant decrease in voice-related QoL, they were considered to be too limited for this study, whereas they represent disadvantages specifically due to dysphonia [10, 12, 14, 22]. In future analysis, it could be considered to include the PL, the second part of the DT&PL. It might be beneficial to differentiate based on the nature of distress.

### Strengths

Regarding strengths, this study is, to the best of our knowledge, the first to describe a comparison between quantitative RRP manifestation and patient experience. This kind of knowledge has increased importance, given that prospective studies of immunotherapeutic agents and systemic or local bevacizumab are underway. Another strength of this study is the accuracy of the results, given that four experienced university-based laryngologists assigned Derkay scores. Additionally, a high inter-observer correlation was found among the laryngologists.

### Limitations

A limitation of the study was that only video stills of the larynx were used to assign a Derkay score to every subject. Although only good-quality recordings were included, scoring results could be more accurate if videolaryngoscopy were used. However, this should be done in a prospective study setting to maintain equal quality of all videos, which might not always be the case in a training hospital.

Another limitation of the study is that intra-observer agreement is calculated based on a small number of patients. About 10% of the inclusions was chosen. More inclusions would have been better from a statistical point of view. However, the study would have become logistically more challenging because of the longer time needed to score. In that case the concentration of the observer could have become a confounding factor. We think that a good middle ground has been found with 10%. The uncertainty in the intra-observer variation is clear from the 95% CI.

Altogether, the moderate association of the variables shows that the Derkay score in its current form is not yet applicable for clinical practice decision-making, nor as an outcome measure for therapy studies. This information might support further development of a uniform staging system. An adjustment suggestion to the current form of the Derkay score could be to add more value to the anatomical scores on sites that frequently cause more complaints. The score of the true vocal cord with respect to the epiglottis or aryepiglottic fold might be multiplied. Another adjustment suggestion could be to assign a numerical value to the current *clinical score* in the Derkay assessment, thereby incorporating the patient’s experience into the actual score. A patient well-being score could be added as an alternative, like the DT. Adding extra scores will logically result in a larger score range, making the total score more precise concerning clinical development. This score and its evolution could properly reflect the patients’ clinical course and therapy response.

## Conclusion

This study shows a moderate association between the Derkay score and QoL in RRP patients. Considering that RRP is a benign lesion, the patient’s well-being is of great importance in staging the condition. Considering the limited conformity between the domains in this study, the numerical Derkay score is not yet applicable for outcome measurement in therapy studies. Additional modifications of the score could be considered to improve its reflection of patient complaints.
